# The Role of BMAL1 in Regulating Circadian Rhythms During Airway Remodeling in Asthma

**DOI:** 10.1155/jimr/6983721

**Published:** 2026-06-11

**Authors:** Xiaorong Xiang, Caiming Zhong, Hao Tang

**Affiliations:** ^1^ Department of Respiratory and Critical Care Medicine, Shanghai Changzheng Hospital, Naval Medical University, Shanghai, China, smmu.edu.cn

**Keywords:** airway remodeling, asthma, BMAL1, circadian clock

## Abstract

Asthma is a chronic respiratory disease characterized by airway inflammation, hyperresponsiveness, and remodeling. The circadian clock governs natural 24 h biological rhythms, encompassing physiological, metabolic, and hormonal fluctuations. It regulates key physiological processes like sleep, activity, and feeding, playing a crucial role in maintaining normal respiratory function. However, the mechanisms underlying asthma airway remodeling and its relationship with circadian clock genes remain incompletely understood. Brain and muscle arnt–like 1 (*BMAL1*), a core circadian clock gene, plays a significant role in the proliferation and differentiation of airway stem cells. This review summarizes recent advances in clinically relevant asthma research, including the role of airway remodeling in disease progression. It then focuses on unresolved scientific questions regarding the function of the *BMAL1* gene in asthma pathogenesis, aiming to contribute to the future development of metabolic rhythm‐based strategies as novel targets for asthma diagnosis and treatment.

**Summary**

• Brain and muscle arnt–like 1 (BMAL1) has emerged as a crucial regulator of airway remodeling in asthma, with its expression and rhythmic activity dynamically influencing the progression of airway structural alterations.
• BMAL1 functions in a cell‐type‐specific manner to regulate asthma airway remodeling, with protective roles in myeloid cells and homeostatic roles in structural cells; its dysregulation promotes pathological remodeling.
• Circadian rhythm disruption leads to airway epithelial dysfunction, abnormal contraction and hypertrophy of airway smooth muscle (ASM) cells, excessive deposition of extracellular matrix (ECM) proteins, and persistent airway inflammation, which collectively contribute to the pathogenesis and progression of asthma.
• Maintaining a normal circadian clock benefits asthma treatment.

## 1. Introduction

Asthma is a chronic respiratory disease whose prevalence has surged dramatically in recent decades, imposing a significant burden on healthcare systems. Systematic analyses predict a continued rise, estimating ~275 million (range: 224–330 million) asthma cases globally by 2050, exerting a substantial global impact [1]. Asthma is a recurrent inflammatory disease of the airways. Repeated inflammatory responses lead to airway tissue remodeling (also known as airway remodeling), which is a fundamental pathological feature of asthma (especially chronic asthma). Based on immune response characteristics, asthma endotypes may be broadly regarded as type 2 (T2) and non‐T2 asthma [2]. Inflammation is a key factor in airway remodeling of asthma, and proinflammatory signals activate and induce the infiltration of inflammatory cells, thereby regulating the pathological process of airway remodeling in asthma through inducing pathological changes such as epithelialization of the airway, thickening of airway smooth muscle (ASM), and deposition of the extracellular matrix (ECM) [3]. Airway epithelial cells (AECs) are the core regulatory factors in the occurrence and development of asthma. They serve as the initiation and transmission hubs of inflammatory pathways and also participate in airway remodeling through processes such as epithelial‐mesenchymal transformation (EMT). As the physical barrier of the airway, their functional state directly affects the body’s response to environmental stimuli, pathogen infections, and other triggering factors. The disruption of their biological clock will further exacerbate the circadian pathological changes of asthma [4]. In mouse models, circadian disruption in the airway epithelium has also been shown to exacerbate asthma development, which is attributed to the reduced responsiveness of the airway epithelium to glucocorticoids [5].

Airway remodeling gradually progresses. Due to the dysfunction of airway epithelium, contraction of smooth muscle cells, and inflammation, it often leads to airway obstruction in asthma patients, with symptoms including coughing, shortness of breath, chest tightness, and wheezing sounds. At the same time, damage to the integrity of the airway epithelium, thickening of the basement membrane, and deposition of collagen under the epithelium result in abnormal airway structure and hardness [6, 7]. Therefore, due to abnormal contraction and hypertrophy of smooth muscle, airway hyperresponsiveness (AHR) occurs, inducing life‐threatening airway narrowing or closure, leading to severe asthma [8].

Notably, most asthma patients experience symptom exacerbations in the early morning or at night, closely linked to the circadian clock. Interestingly, studies show that going to bed after 11 PM correlates positively with asthma prevalence [9]. It indicates that the disruption of the circadian rhythm may affect the incidence of asthma. Aryl hydrocarbon receptor nuclear translocator‐like protein 1 (ARNTL or brain and muscle arnt–like 1 [BMAL1]) is a circadian transcriptional activator. BMAL1 is also widely expressed in peripheral tissues and plays a vital role in organ and tissue metabolism [10]. One retrospective case‐controlled study shows that among patients with poorly controlled asthma, the expression of circadian clock genes *BMAL1*, *CK1*ε, *PER1*, and *PER2* was significantly altered in those with nocturnal symptoms compared to those without [11]. Nevertheless, existing research is insufficient to fully explain the circadian nature of asthma and requires further investigation. The changes in lung function indicators of asthma patients show a significant diurnal fluctuation pattern in relation to clinical symptoms such as wheezing and chest tightness. This phenomenon has been confirmed by numerous clinical studies and basic experiments, and it is a consensus conclusion in this field [12]. However, up to now, the specific causal regulatory mechanism between the circadian rhythm regulation and the onset and symptom fluctuations of asthma has not been fully elucidated. At the same time, there are still many unclear issues regarding the clinical stratification of asthma patients based on the circadian rhythm and the formulation of individualized treatment plans, which require further in‐depth exploration. Therefore, there is an urgent need for updated evidence to explore whether the disruption of the circadian rhythm will aggravate the occurrence of asthma, and the underlying mechanism still requires further investigation.

Therefore, this review specifically focuses on the diurnal variation of asthma symptoms and the role of BMAL1 in regulating airway remodeling changes. It highlights recent advances, emphasizing BMAL1’s function in asthma airway remodeling, to provide insights for potential future therapeutic interventions.

### 1.1. Literature Search Strategy

To ensure the rigor, comprehensiveness, and credibility of this review, especially for the strong claims regarding the immune mechanisms driving asthma airway remodeling and the regulatory role of BMAL1, a systematic literature search was conducted following standardized guidelines. The detailed search strategy is described as follows. Three authoritative international databases were selected to cover relevant studies comprehensively, including PubMed, Web of Science, and Scopus. The literature search was conducted with a time range spanning from January 2000 to January 2026 to include both classic foundational studies and the latest research advances in the field of asthma airway remodeling, circadian rhythm, and immune pathways. Key search terms were combined using Boolean operators to ensure the accuracy and comprehensiveness of the search results. The combination strategy is as follows: (“Asthma” OR “bronchial asthma”) AND (“Airway remodeling” OR “airway structural remodeling”) AND ((“BMAL1” OR “ARNTL”) AND (“Circadian clock” OR “circadian rhythm” OR “circadian regulation”)) OR (“Asthma” OR “bronchial asthma”) AND (“Airway remodeling” OR “airway structural remodeling”) AND (“type‐2 inflammation” OR “eosinophils” OR “immune pathway”).

The included literature must meet the following criteria to ensure the scientificity and relevance of the review. Original research articles, review articles, and clinical trials that focus on the association between immune cells/pathways, circadian clocks (BMAL1), and asthma airway remodeling. Studies were published in English with full‐text availability to ensure the accessibility of detailed data and research methods. Studies conducted in human subjects, animal models (e.g., BMAL1‐knockout mice and asthma mouse models), or in vitro experiments (e.g., human AECs and ASM cells) provide direct or indirect evidence supporting the regulatory roles of immune components and BMAL1 in airway remodeling. Research content: Studies that address immune‐mediated remodeling mechanisms or the interaction between circadian rhythm and asthma airway remodeling. The following types of literature were excluded to avoid irrelevant or low‐quality data: case reports, letters to the editor, abstracts without full text, and conference proceedings. Studies were unrelated to immune regulation, circadian rhythm, or asthma airway remodeling. Studies with incomplete data, unclear research design, or poor methodological quality cannot support the core assertions of this review. The flow diagram of the literature selection is presented in Supporting Information Figure S1. The references cited are consistent with the literature sources of the original review, ensuring academic continuity and credibility.

## 2. The Circadian Clock in Asthma

### 2.1. Circadian Clock

The mammalian circadian system drives daily rhythms in physiology and behavior [13]. Circadian rhythms are generated at the cellular level by autoregulatory feedback loops of interconnected transcription factors (collectively termed clock genes). Besides the central clock in the suprachiasmatic nucleus of the hypothalamus, peripheral tissues, including the lung, liver, heart, and kidneys, also contain cell‐autonomous oscillators [14]. Circadian disruption refers to the disturbance of the timing system and/or the daily rhythm of the molecular clock. Any significant change in the circadian timing or amplitude of molecular clock genes that affects the expression of downstream clock‐controlled output genes or cellular processes can be termed clock dysfunction [15]. The core circadian mechanism forms a loop involving core transcription factors, including BMAL1 and circadian locomotor output cycles kaput (CLOCK). These form a heterodimer acting as the primary positive limb of the clock loop, which regulates the expression of period (PER) and cryptochrome (CRY) [16]. At the beginning of the circadian rhythm, the spiral‐ring‐spiral transcriptional regulatory factors CLOCK and BMAL1 form a heterodimeric complex, which subsequently promotes chromatin remodeling and activates the expression of three *PER* and two *CRY* genes through E‐box DNA regulatory sequences [17]. Then, the PER and CRY protein complexes formed by transcription and translation accumulate in the cell nucleus, thereby inhibiting the activation of the E‐box caused by CLOCK and BMAL1. When *PER* and *CRY* are degraded and cleared in the cell nucleus, CLOCK and BMAL1 are reactivated, completing one cycle. PER and CRY in turn form an autoregulatory transcriptional–translational feedback loop to regulate CLOCK/BMAL1 [18]. Auxiliary systems include two competing nuclear receptor families: nuclear receptor subfamily 1 group D (NR1D), also known as Rev‐erb, and retinoic acid receptor–related orphan receptors (RORs). RORs directly activate *BMAL1* gene transcription, while Rev‐erb acts as its repressor, linking the immune system and the clock [19, 20]. Furthermore, clock oscillations in Rev‐erbα ligands (heme and carbon monoxide) may influence not only the phase and amplitude of circadian rhythms but also the physiological outputs of the circadian system. Thus, Rev‐erbα may participate in synchronizing central and peripheral clocks [21–23].

### 2.2. Asthma

**Figure 1:**
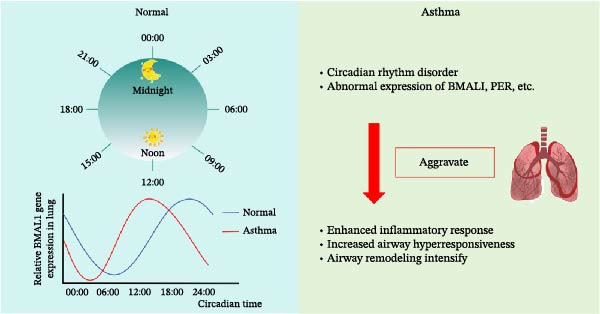
Asthma biological clock. In healthy individuals (left panel), physiological circadian rhythms entrained by light maintain stable airway homeostasis. In asthma (right panel), normal circadian rhythmicity is exaggerated, leading to pronounced nighttime worsening of airway obstruction, increased inflammatory cell infiltration, and airway inflammation.

Asthma is an inflammatory airway disease with distinct circadian characteristics, featuring intermittent bronchoconstriction, excessive mucus secretion by bronchial epithelial goblet cells, airway remodeling, and chronic airway inflammation. Airway inflammation and constriction follow circadian patterns, with symptoms often worsening at night. Most asthma attacks occur late at night or early morning, severely impacting patients’ sleep quality [24–26]. The circadian exacerbation of asthma symptoms was reported long ago [27], and recent evidence increasingly describes the asthma‐circadian relationship, showing that circadian disruption may contribute to asthma development [28–30]. Night shift work causes misalignment between endogenous circadian timing and the external light/dark cycle and is associated with metabolic disorders and cancer. Studies indicate that individuals chronically exposed to night shift work have a significantly higher risk of moderate‐to‐severe asthma, with a significant dose‐dependent relationship between extended duration/frequency of night shifts and increased asthma incidence [31, 32]. Meta‐analyses also show a significant association between nocturnal light exposure and asthma occurrence [33]. While direct links between circadian rhythms and asthma control/quality of life are yet to be fully explored, recent research reports that asthma patients with a morning chronotype (early risers) experience a healthier quality of life [34]. Collectively, this suggests that maintaining a normal circadian clock may be beneficial for asthma management (Figure 1). Several theories propose mechanisms for nocturnal asthma worsening, such as abnormal number/function of nocturnal β2–adrenergic receptors, decreased cortisol levels, and increased melatonin levels potentially exacerbating airway inflammation [25, 35]. However, these theories remain speculative, and the precise mechanisms underlying nocturnal symptom exacerbation are still unclear.

### 2.3. Link Between Circadian Clock and Asthma

The seminal paper by Scheer et al. [36] utilized three complementary experimental designs to conclusively demonstrate for the first time in humans that airway obstruction is independently regulated by endogenous circadian rhythms. After eliminating the interference of behavioral and environmental factors such as sleep, posture, and activity, the study isolated the independent influence of circadian rhythms on lung function. This provided crucial physiological evidence for the mechanism of nocturnal fluctuations in asthma. The core finding showed that airway resistance and forced expiratory volume in 1 s (FEV_1_) of asthma patients followed prominent circadian oscillations: during the PER spanning approximately 11 p.m. to 11 a.m. on the subsequent day, lung function declined to its minimum synchronously with the nadir of core body temperature. The peak airway resistance was 20% higher than during the day, and the FEV_1_ trough was 7% lower than during the day. Moreover, the probability of using rescue bronchodilators was four times that of the daytime. Although healthy controls also exhibited similar rhythms, the amplitude was significantly lower (FEV_1_ fluctuation was only 3%), confirming that this rhythm was amplified in asthma patients. It indicates that the circadian rhythm disorder in asthma patients is closely related to AHR, ASM function, and inflammatory signaling pathways [37]. The changes in airway function may be related to abnormal circadian rhythms causing abnormal airway diameter and increased airway resistance, which in turn lead to respiratory symptoms [38].

**Figure 2:**
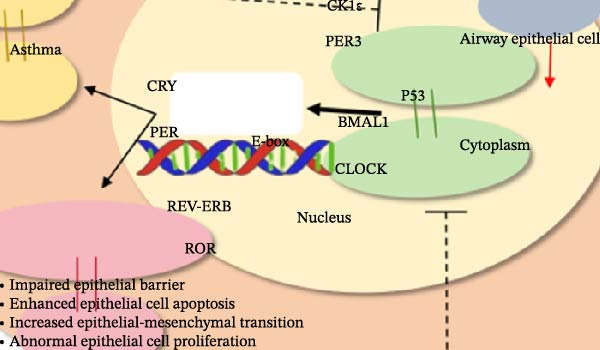
The molecular circadian clock (Note: BMAL1, brain and muscle arnt–like 1; CLOCK, circadian locomotor output cycles kaput; CRY, cryptochrome; PER, period; RORs, retinoic acid receptor–related orphan receptors). In asthma, dysregulation of the core circadian clock (including reduced CK1ε/PER3 and impaired p53‐mediated BMAL1 regulation) disrupts airway epithelial homeostasis, leading to barrier impairment, apoptosis, EMT, and abnormal proliferation, ultimately promoting airway inflammation and asthma progression.

Oishi and colleagues first detected clock gene expression in the lung [39], later confirmed by Gibbs et al. [40] regarding functional molecular clocks in the bronchial epithelium. Current studies have revealed that the volatile organic compounds (VOCs) exhaled by asthma patients exhibit significant diurnal fluctuations, and the administration time of inhaled glucocorticoids alters the rhythmic characteristics of VOCs. The rhythmic changes of characteristic VOCs such as methylthioacetic acid and monoterpene are related to the diurnal patterns of asthma inflammation [41]. Meanwhile, these studies confirmed that the rhythmic expression of the *PER3* gene in the peripheral blood of patients with asthma was significantly elevated, with a markedly increased amplitude [42, 43]. Furthermore, Powell et al. further validated the abnormally increased amplitude of the *PER3* gene rhythmicity in primary AECs from children with asthma. This alteration is independent of systemic signals and represents an intrinsic circadian disorder of the airway epithelium [44]. Analysis of core clock genes in peripheral blood from asthmatic patients revealed significantly reduced expression of *BMAL1* and *CK1*ε in patients with poor asthma control and downregulated *PER3* expression in patients with nocturnal symptoms [11]. In summary, circadian clock gene expression is altered in patients with bronchial asthma (Figure 2).

Research into pulmonary circadian rhythms continues to evolve. While some studies have described potential mechanisms involving clock genes in asthma development, limitations in these studies mean that understanding how circadian disruption exacerbates asthma airway remodeling remains elusive.

## 3. The Role of BMAL1 in Asthma Airway Remodeling

**Figure 3:**
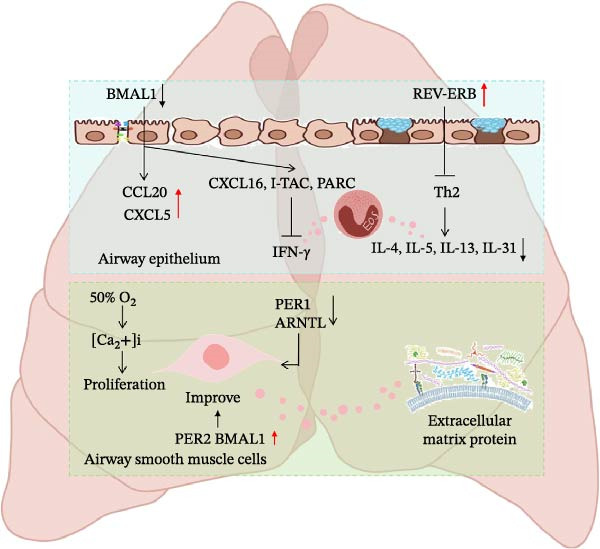
Disrupted biological clock aggravates airway remodeling in asthma (Note: ARNTL, aryl hydrocarbon receptor nuclear translocator‐like protein 1; BMAL1, brain and muscle arnt–like 1; CCL2, monocyte chemokine ligand 2; CLOCK, circadian locomotor output cycles kaput; CXCL5, neutrophil chemokine ligand 5).

Mounting evidence indicates that the circadian clock is crucial in lung physiology and disease. Airway remodeling is a core pathological feature of asthma, characterized by sequential and interconnected structural alterations in AECs, ASM cells, and ECM deposition. *BMAL1*, as a core circadian clock gene, regulates these processes in a cell‐type‐specific manner, and its dysregulation exacerbates pathological remodeling (Table 1). The pathological features of airway remodeling across all airway sizes include goblet cell metaplasia, subepithelial fibrosis, and ASM hyperplasia or hypertrophy, which collectively drive structural dysfunction [54] (Figure 3).

**Table 1:** BMAL1‐mediated regulation in asthma airway remodeling.

Experimental setting	Subject	Research model	Effect	References
In vivo	Human	Leukocyte derived from patients with asthma	Downregulation of BMAL1 expression	[11]
		AECs derived from patients with asthma	Abnormalities in the circadian gene rhythm Disordered neutrophil chemotaxis pathway	[44]
		PBMC and MDM derived from patients with asthma	Disruption of the circadian rhythm Inflammation regulation imbalance	[45]
		Eosinophil derived from patients with asthma	Disruption of cellular rhythms Downregulation of BMAL1 expression IL‐5 proinflammatory effects	[46]
	Mouse	BMAL1‐knockout mice induced by HDM	Abnormal expression of chemokines Airway collagen deposition Glucocorticoid resistance	[5]
		BMAL1‐knockout mice induced by LPS	Neutrophils migrate into the lungs Resistance to glucocorticoids increases	[40]
		BMAL1‐knockout mice induced by OVA and PM2.5	Increase of P53 protein Stimulate autophagy Lead to airway remodeling	[47]
		BMAL1‐knockout mice induced by LPS	Disruption of the circadian rhythm Exacerbating inflammatory invasion	[48]
		Rev‐erbα–knockout mice induced by LPS	Increase of neutrophil infiltration	[49]
In vivo	Mouse	BMAL1‐knockout mice	Increased airway resistance Changes in airway structure Impaired lung function	[50]
		BMAL1‐knockout mice induced by moderately hyperoxic	Increased airway hyperresponsiveness Abnormal regulation of intracellular calcium concentration Airway structural remodeling	[51]
		BMAL1‐knockout mice induced by OVA	Increased lung inflammation Eosinophil infiltration High expression of Th2‐type inflammatory factors	[52]
		Mice induced by HDM	Disruption of the circadian rhythm Inflammation regulation imbalance	[45]
In vivo	Mouse	Mice induced by HDM	Disruption of cellular rhythms Downregulation of BMAL1 expression IL‐5 proinflammatory effects	[46]
		Rev‐erbα–knockout mice induced by HDM	Decreased expression of BMAL1 Impaired pulmonary circadian rhythms	[53]
In vitro	AEC	Asthma induced by HDM	Abnormal expression of chemokines Airway collagen deposition Glucocorticoid resistance	[5]
		BMAL1‐knockout	Disruption of the circadian rhythm Exacerbating inflammatory invasion	[48]
	NHBE	IL‐1β–stimulated NHBE cell model	Neutrophils migrate into the lungs Resistance to glucocorticoids increases	[40]
		BMAL1‐knockout	Increase of P53 protein Stimulate autophagy Lead to airway remodeling	[47]

Abbreviations: AEC, airway epithelial cell; MDMs, monocyte‐derived macrophages; NHBE, normal human bronchial epithelial cells; PBMC, peripheral blood mononuclear cell.

Repeated shifts in environmental light schedules (e.g., jet lag or shift work) are known to disrupt circadian activity and overall rhythmicity, leading to increased lung tumor formation in p53‐knockout mice [55]. Recent research indicates that low *BMAL1* expression contributes to airway remodeling in PM2.5‐exposed asthmatic mice. Although BMAL1 knockdown did not alter p53 mRNA levels in AECs, it affected p53 protein stability by directly binding to p53, reducing its proteasomal degradation and promoting p53 protein accumulation [47].

### 3.1. Airway Remodeling

#### 3.1.1. AEC

AECs, as the core regulatory factors of asthma inflammation and airway remodeling, have a crucial role in the occurrence and development of the disease due to their disrupted circadian rhythms [56]. Circadian components interact with key elements of epithelial barrier function and immune responses, regulating biological processes on a 24 h cycle under homeostasis. This likely represents an anticipatory defense against harmful day/night stimuli like environmental pathogens. As we know, rhythm disruption can trigger allergic diseases like asthma by altering the epithelial barrier and immune function [57]. As a key initiator in asthma pathogenesis, AECs produce cytokines capable of disrupting lung homeostasis and promoting airway remodeling, including eotaxin‐1 and TGF‐β1 [58]. Local circadian rhythms in lung AECs are crucial for pulmonary homeostasis, coordinating major cellular processes like metabolism and immunity.

Based on the study of primary AECs from children with asthma, it was shown that the overall periodicity and rhythmic maintenance of the core circadian genes were intact, but the *PER3* gene exhibited a significantly increased amplitude of expression change, and this change was independent of systemic signals and belonged to the inherent rhythmic abnormalities of epithelial cells [44]. At the same time, the rhythmic disorder of the *PER3* gene was closely related to the rhythmic abnormalities of the neutrophil chemotaxis pathway, further exacerbating the diurnal fluctuations of airway inflammation [40]. Another study also confirmed that the rhythmicity of the *PER3* gene was enhanced in the peripheral blood of asthma patients, which was consistent with the results of the study on AECs, suggesting the altered expression of the *PER3* gene of the abnormal circadian rhythm in asthma [43]. Another study shows that a Rev‐erb agonist can attenuate T helper 2 cell (Th2) cytokine–induced epithelial barrier dysfunction in human bronchial epithelial cells, reducing airway epithelial permeability, increasing transepithelial electrical resistance, and improving mRNA and protein levels of selected epithelial barrier and circadian target genes [59]. This indicates the significant potential of targeting specific circadian genes to protect the airway epithelial barrier function and suggests therapeutic implications for asthma.

#### 3.1.2. ASM Cell

ASM dysfunction is a key factor in airway narrowing in asthma, characterized by excessive secretion of inflammatory factors, increased mass, and enhanced contractile responses. These pathological features play significant roles in propagating airway inflammation, structural remodeling, and escalating AHR and are major limitations of current therapeutic strategies [60]. The proinflammatory environment in asthma affects the ASM structure and function. ASM expresses a range of inflammatory receptors, which, upon activation, contribute to hallmark asthma features, particularly immune cell recruitment/activation, hypercontraction, proliferation, migration, and ECM protein deposition [61]. Ehlers et al. [50] reported that BMAL1 deletion in mice worsened acute viral bronchiolitis caused by the influenza A virus. Additionally, BMAL1‐knockout mice exhibited asthma‐like airway changes postinfection, including increased airway resistance, and it remains to be further explored whether this is related to ASM [50]. In a mouse model of moderate hyperoxia, neonatal mice exhibited increased AHR and alterations in ASM, leading to heightened airway tone, impaired airway dilation, and structural remodeling. Mechanistically, the ASM cell clock is highly relevant to asthma pathophysiology; deletion of the core clock component Bmal1 increased inflammation and significantly worsened lung function under hyperoxia [51]. Moderate hyperoxia (50% O_2_) enhanced intracellular calcium ([Ca_2_+]i), promoting the proliferation of human fetal ASM, thereby contributing to bronchospasm and remodeling. Disrupting the ASM clock by inhibiting PER1 or BMAL1 (ARNTL) altered [Ca_2_+]i regulation under varying O_2_, promoting calcium influx. Conversely, overexpressing *PER2* and *BMAL1*, driving the clock protein expression, rescued bronchial remodeling [62]. In mice lacking the Rev‐erbα clock gene, the circadian effect on hyperresponsiveness was eliminated, and rhythmic expression of key muscarinic receptor subtypes mediating cholinergic smooth muscle responses disappeared [53]. This suggests that the circadian effect on asthma responses is mediated by the circadian clock through changes in airway reactivity, smooth muscle tone, and airway narrowing.

Increased deposition of ECM proteins in the airway wall, particularly in the reticular basement membrane, is an early and characteristic feature of severe asthma [63]. ECM changes contribute to airway inflammation, remodeling, ASM function, and the characteristic morphology in asthma [64]. Research shows that BMAL1 knockout increases collagen deposition near large airways in 1‐year‐old mice, a long‐term consequence of increased neutrophil infiltration [48]. Another study found that under moderate hyperoxia, BMAL1 knockout worsened airway resistance, reduced lung compliance, and increased ECM deposition in mice, adversely affecting lung development [51]. The ECM is the main determinant of the structure and mechanical behavior of the lung. However, in the asthmatic lung, the matrix seems to contribute to airway inflammation, airway remodeling, and those alterations of the smooth muscle function of the airway and morphology typical of asthma. Therefore, maintaining the structure and morphology of the normal ECM may be beneficial for the improvement of asthma.

### 3.2. Airway Inflammation

The rhythmic changes of inflammatory‐related indicators during rhythm disorder include eosinophil recruitment, goblet cell hyperplasia, excessive release of T2 inflammatory factors, etc. These may further exacerbate Th2‐type inflammation by enhancing airway mucosal inflammation [65]. Airway remodeling in asthma is a complex and persistent pathological process that is not only shaped by structural changes in compartments such as the epithelium, ASM, and ECM but also tightly regulated by a variety of immune cells and their mediated signaling pathways [66]. T2 inflammatory response is the core immune mechanism driving airway remodeling in most asthmatic patients, in which T2 cytokines, T2 innate lymphoid cells (ILC2s), Th2 cells, eosinophils, and antigen‐presenting cells (APCs) form a coordinated regulatory network to promote structural remodeling of the airway [67].

#### 3.2.1. Adaptive Immunity

Under normal physiological conditions, key processes of adaptive immunity such as cellular activation and cytokine secretion are precisely regulated by the circadian clock. In the pathological progression of asthma, however, circadian rhythm disruption exerts a prominent regulatory effect on the response patterns of adaptive immunity [49, 68]. Notably, the dysregulation of adaptive immunity centered on the Th2‐type immune response is closely associated with the persistence of airway inflammation and the progression of airway remodeling in asthma.

**Figure 4:**
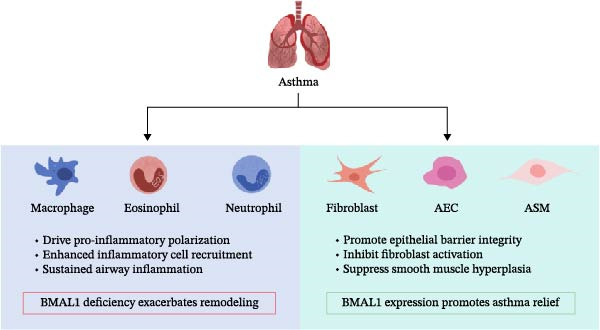
Dual roles of BMAL1 in regulating airway remodeling in asthma by cell type. BMAL1 exerts opposing effects on airway remodeling depending on cell type. In immune cells, BMAL1 deficiency promotes inflammation and exacerbates remodeling. In structural cells, BMAL1 expression maintains epithelial barrier integrity and inhibits fibroblast/smooth muscle activation, alleviating remodeling and asthma progression.

IL‐5, mainly secreted by ILC2s and Th2 cells, specifically promotes the maturation, activation, and survival of eosinophils. Activated eosinophils release toxic granule proteins, which induce epithelial cell injury, stimulate fibroblasts to secrete ECM, and further accelerate airway fibrosis and remodeling. A previous study demonstrated that activation of the IL‐5 signaling pathway in fibroblasts from asthmatic tissues can trigger ECM imbalance and inhibit cellular apoptosis, thereby further exacerbating airway remodeling [69]. Research results show that the circadian rhythm of eosinophils in asthma patients is disordered, and the expression of BMAL1 is lower in patients compared to healthy individuals. By upregulating the expression of BMAL1, the aggregation of airway inflammatory cells can be reduced, the recruitment of eosinophils can be decreased, and the proinflammatory effect of IL‐5 can be antagonized (Figure 4) [53]. Zaslona et al. [70] used mice lacking BMAL1 in myeloid cells to establish an ovalbumin‐induced asthma model and found significantly exacerbated asthma features, including increased lung inflammation, evidenced by substantial increases in eosinophil numbers in lungs and serum and elevated IL‐5 levels, suggesting the circadian protein BMAL1 acts as a potent negative regulator of allergic asthma. This finding may explain the increased nocturnal asthma cases associated with low BMAL1 expression.

BMAL1 is essential for maintaining normal human circadian rhythms as a key circadian regulator. BMAL1 knockout disrupts the circadian expression of inflammatory chemokines, promoting neutrophil chemokine ligand 16 (CXCL16), I‐TAC, and PARC expression while significantly suppressing IFN‐γ expression, thereby exacerbating asthma symptoms in mice [52]. Other T2 cytokines like IL‐4 could promote B cell class switching to produce IgE, enhance the adhesion and recruitment of inflammatory cells, and induce the proliferation and migration of ASM cells; IL‐13 can directly stimulate AECs to secrete ECM components, promote EMT, reduce epithelial barrier function, and simultaneously induce ASM hypertrophy and hyperplasia, leading to airway narrowing [71]. Biologics targeting T2 inflammation have been widely used in clinical practice. Anti‐IL‐5 drugs (such as mepolizumab) can reduce eosinophil infiltration, anti‐IL‐13 drugs (such as lebrikizumab) can inhibit the profibrotic effect of IL‐13, and anti‐IgE drugs (such as omalizumab) can block the activation of allergic reactions, all of which can effectively delay or reverse airway remodeling in asthmatic patients by targeting key links of immune pathways [72].

Interestingly, a research showed the melatonin treatment further increased the OVA‐induced expressions of PER1 and CLOCK without affecting other circadian clock protein expression; however, melatonin receptor antagonist luzindole remarkably decreased the OVA‐induced expressions of PER1, BMAL1, CRY1, and CRY2 while further increasing the OVA‐induced expression of Timeless [73]. These results demonstrate that the altered expression of circadian clock proteins in allergic lung tissue is closely associated with pulmonary inflammatory responses, with distinct clock components exerting differential regulatory effects—some clock proteins may mitigate lung inflammation when their expression is modulated, while aberrant expression of others could potentially exacerbate inflammatory processes.

#### 3.2.2. Innate Immunity

In the pathological progression of asthma, circadian rhythm disruption exerts a profound and pervasive impact on innate immunity, the first line of the body’s immune defense against exogenous stimuli and pathogenic invasion. The immune system is under strong circadian control [74]. Circadian rhythm dysregulation not only undermines the innate immune barrier function of the airway, making it more susceptible to allergen and pathogen stimulation, but also initiates and amplifies the persistent airway inflammatory cascade.

The rhythmicity of the clock‐controlled immune system significantly impacts the development of infectious and inflammatory diseases [75, 76]. Toll‐like receptor 9 (TLR9), a key pattern recognition receptor (PRR) anchored in the endosomal membrane of innate immune cells, is a critical mediator of innate immune responses in asthma [77]. The BMAL1/CLOCK heterodimer regulates TLR9 expression and lowers levels of inflammatory monocyte chemokine ligand 2 (CCL2), while the auxiliary clock component Rev‐erbα inhibits interleukin‐6 secretion [78]. Antigen presentation is a key initiator of the immune response that drives airway remodeling. Macrophages, as an important type of APC, can be polarized into M2 macrophages under the induction of T2 cytokines and secrete IL‐10, TGF‐β, etc., to promote fibroblast proliferation and ECM deposition, participating in airway fibrosis remodeling. Nevertheless, lung macrophages are instrumental in protecting against pathogens and play a critical role in the resolution of inflammation and return to homeostasis [79]. A study showed that the expression of BMAL1 protein in macrophages of asthma patients reached its peak at 20 o’clock, and the expression of CLOCK protein reached its peak at 16 o’clock, showing a different trend from healthy samples. The oscillatory expression of other proteins Rev‐erb and ROR also changed significantly, suggesting that the molecular circadian clock interacts with the infiltration of monocytes/macrophages in asthmatic airways [45].

At present, there is a great deal of interest in neutrophilic asthma. An increase in the number of neutrophils or a decrease in their apoptosis can both lead to airway inflammation [80]. One of the core pathological features of nocturnal asthma is the diurnal fluctuation of inflammatory cells in the airways. Human studies have confirmed that the content of neutrophils and eosinophils in the airways of asthma patients reaches its peak in the early morning [81], and this rhythm is highly consistent with the nocturnal low peak of cortisol levels, suggesting that the recruitment of inflammatory cells is closely related to the regulation of the circadian rhythm [82].

The human AEC study by Powell et al. [44] further revealed that the rhythmicity of the neutrophil chemotaxis–related pathways in the AECs of asthma patients was significantly changed, and the chemokines such as CXCL2 and CXCL5 showed rhythmic expression with abnormally elevated amplitude, and these chemokines are the key molecules mediating the infiltration of neutrophils into the airways. Animal experiments also verified this regulatory relationship [48, 83]. At the molecular level, research identified a pathway linking the circadian clock (via BMAL1 and Rev‐erbα) to the regulation of the airway epithelial glucocorticoid receptor, thereby controlling neutrophilic inflammation [40, 49, 84]. Glucocorticoids, the first‐line antiasthma drugs, mainly inhibit the activation of ILC2s, Th2 cells, and eosinophils; reduce the secretion of T2 cytokines; and thereby alleviate airway inflammation and remodeling [85]. However, some patients with severe asthma show steroid resistance [86]. Therefore, a variety of biological agents have played a significant role; however, asthma biological agents still have limitations in target coverage and unclear long‐term efficacy. Individualized combination strategies need to be explored. In the future, more precise and broad‐spectrum new formulations should be developed by integrating multiomics and clock genes to optimize clinical benefits.

## 4. Interaction Between BMAL1 and Hypoxia in Asthma Airway Remodeling

The circadian clock and oxygen‐sensing pathways are interconnected [87]. In lung disease, circadian clock dysfunction may be an important early consequence of hypoxia‐driven diseases and contribute to downstream processes [88]. The mechanism may involve core clock genes altering expression levels under hypoxia, while pulmonary oxygen levels, dependent on hypoxia‐inducible factor (HIF‐1α) activation, synchronize the cellular clock. HIF‐1α transcriptionally controls the core clock gene expression. A bidirectional relationship exists where HIF‐1α heterodimerizes with BMAL1 to regulate downstream targets, and BMAL1 regulates HIF gene expression in relation to the circadian clock. In BMAL1‐deficient cells, elevated HIF‐1α levels and reduced NRF2 activity lead to increased production of the proinflammatory cytokine IL‐1β. HIF can also be upregulated by hyperoxia, highlighting cellular sensitivity to changing O_2_ levels [89–93].

Further exploring the mechanism, results indicate that hypoxia response and circadian pathways are enriched among upregulated genes in endothelial cells, myofibroblasts, and AT2 cells responding to intermittent hypoxia (IH) [94]. DAVID enrichment analysis of the top 200 up‐ and downregulated genes in hypoxic‐exposed mice revealed significantly associated pathways including the circadian rhythm, angiogenesis, and ECM organization. Furthermore, upregulation of various ECM‐related pathways in fibroblasts (e.g., ECM proteoglycans, nonintegrin membrane‐ECM interactions, and collagen chain trimerization) may relate to FGF signaling pathway activation in myofibroblasts during IH [95].

Recent findings show that rhythmic oxygen changes in vitro reset the clock in a HIF‐1α–dependent manner, emphasizing the power of the O_2_‐clock relationship [51]. A study on the treatment of mice under low‐oxygen conditions revealed that in the brain tissue of mice, the expression levels of core circadian clock genes per1 and clock were significantly correlated with the oxygen concentration. Moreover, these hypoxic effects might be regulated by HIF‐1α, indicating that hypoxia does indeed affect the operation of the biological clock [96]. Another study found that under moderate hyperoxia, BMAL1 knockout worsened airway resistance, reduced lung compliance, and increased ECM deposition in mice [21]. Oxygen‐sensing pathways and circadian rhythms are evolutionarily conserved adaptations to enable cells and organisms to respond to a changing environment [97]. The oxygen environment is a key regulatory factor for the circadian rhythm disorder of asthma. During the fetal PER, low oxygen levels interact with the circadian rhythm through HIF‐1α in a bidirectional manner, participating in lung development; after birth, exposure to high oxygen levels may disrupt the oscillation of clock genes such as Rev‐erbα, affecting the maturation and function of ASM [87]. However, further research is needed to explore how the oxygen‐sensing pathway can exert its physiological properties in lung tissue, with the aim of enabling a deeper understanding of the pathogenesis of asthma in the future.

## 5. Conclusion and Perspectives

**Figure 5:**
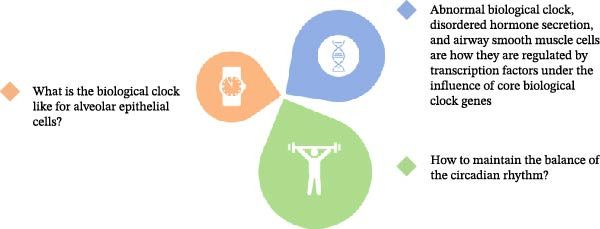
Diagram of the pending issue in targeting biological clock as an asthma therapy.

Existing research confirms that a common feature of chronic airway diseases is disruption of the daily/circadian rhythm in lung function and inflammatory responses [98], suggesting that understanding asthma pathogenesis and interventions from a circadian perspective is promising. However, due to the diversity and complexity of circadian rhythms, many questions regarding the exact mechanisms in asthma require further exploration. First, the initial step in airway remodeling involves the dysfunctional airway epithelium. While current research focuses on circadian rhythms in AECs, alveolar epithelial cell transformation plays a significant role in asthma. Yet, circadian rhythms in alveolar epithelial cells remain poorly understood—should their clocks be investigated? This needs ongoing study. Second, asthma symptom worsening at night is currently linked to cortisol imbalance, melatonin abnormalities, and circadian disruption. Since circadian disruption often causes hormonal secretion abnormalities, the mechanisms by which clock disruption and hormonal dysregulation worsen asthma symptoms and how ASMCs and core clock genes lead to nocturnal abnormal contraction under specific transcriptional regulation remain unknown. How to regulate the expression of the core protein BMAL1‐CLOCK complex and thereby influence the biological clock is an attractive intervention point. Current research has identified a small molecule drug, the core circadian rhythm regulator (CCM), which can target the cavity of the PASB domain of BMAL1, causing it to expand, resulting in a conformational change in the PASB domain and altering the function of *BMAL1* as a transcription factor, making it possible to directly access the cellular activity of BMAL1‐CLOCK, and also diversifying and enhancing the precision of regulating the circadian rhythm [99]. Third, while maintaining a normal circadian clock benefits asthma treatment, how is circadian balance best achieved (Figure 5)? Recent research on intermittent fasting reversing circadian disruption is noteworthy. Nighttime feeding exacerbated LPS‐induced lung inflammation in mice, including increased BAL total white blood cells, neutrophil infiltration, and CXCL5 levels. Time‐restricted feeding reversed the expression of core clock genes like *BMAL1* [55]. Therefore, the lung clock might synchronize with exogenous feeding‐related signals, suggesting a strategy for preventing/treating allergic diseases by aligning endogenous clocks with exogenous environmental cycles. Interestingly, therapies based on the molecular clock and asthma inflammation, termed “chronotherapy,” are emerging. Multiple studies consistently indicate that the optimal time for asthma medication administration is afternoon or evening, not morning [100]. However, asthma attacks often peak at night and early morning—why this discrepancy between optimal dosing time and symptom onset? The specific mechanisms of “chronotherapy” require further investigation.

A clinical trial shows that, when inhaled corticosteroids, fluticasone furoate, were administered separately to asthma patients in the morning and at night, the results showed that both lung function and asthma symptoms improved, and the effects were comparable [101]. Asthmatic mice with different circadian rhythms from humans were treated with dexamethasone. The results showed that at 0 o’clock, when the mice were resting, the expression level of the calcium‐activated chloride channel regulator (*CLCA3*) gene that promotes excessive mucus secretion could be reduced. This suggests that administering the drug before rest might be able to change the symptoms. Corresponding to the human biological clock, administering the drug at night might be beneficial [102]. Due to the inconsistency between animal experiment results and clinical trial results, there is currently no unified time‐based treatment plan for asthma. Therefore, it is not recommended to change the dosing regimen based on the existing experimental studies. Instead, the clinical trial results and reports on the timing of the day should be used as important key variables [103].

It is of great significance that there are significant differences in the phase distribution, core functional association, and molecular mechanistic compatibility of the circadian rhythm regulation between nocturnal organisms (mice) and diurnal organisms (humans) [104, 105]. In mice, the expression rhythm of core clock genes (BMAL1; PER1, 2, 3; and CRY1, 2) is synchronized with the activity PER. BMAL1, as a positive regulatory factor, reaches its peak expression at night (activity peak), driving metabolic and immune pathways to adapt to nocturnal foraging and defense behaviors. PER/CRY and other inhibitory factors peak during the day (rest PER), maintaining the rhythmic stability [106]. In humans, the oscillation phase of core clock genes is opposite to that of mice. The peak expression of BMAL1 is located during the day (activity peak), while PER/CRY factors are enriched at night (rest PER), ensuring that physiological functions match daytime work, eating, and other behaviors. This phase reversal results in a complete inversion of the functional effects of the same clock genes on the diurnal dimension [107]. These differences suggest that when translating the research results related to the circadian rhythm of mice to humans, it is necessary to first correct the deviations caused by the phase shift, and the conclusions from animal experiments cannot be directly applied.

Given the strong link between asthma occurrence and the high prevalence of night shift work, impacting both individual health and public health [29, 108], future longitudinal follow‐up studies are needed to determine if adjusting shift schedules based on chronotype could serve as a public health measure to reduce the risk of inflammatory diseases like asthma.

## Author Contributions

**Xiaorong Xiang**: writing – original draft, data collection, formal analysis, figures and table sorting. **Caiming Zhong**: writing – review, editing, supervision. **Hao Tang**: writing – review, editing, funding acquisition.

## Funding

This work was supported by the National Natural Science Foundation of China (Grant 82070036) and the “Deep Blue” Engineering “Strong Sea” Innovation Team Project.

## Consent

The authors have nothing to report.

## Conflicts of Interest

The authors declare no conflicts of interest.

## Supporting Information

Additional supporting information can be found online in the Supporting Information section.

## Supporting information


**Supporting Information** Figure S1: The literature selection flow for this review. A total of 1352 records were retrieved from PubMed, Web of Science, and Scopus. After removing 830 duplicates, 522 records remained. Irrelevant studies were excluded by title screening, leaving 104 records; two additional records were excluded by abstract screening, leaving 102 full‐text articles for eligibility assessment. After further exclusion based on patient, article type, intervention, and full‐text unavailability, 16 reviews and 70 original studies were finally included.

## Data Availability

Data sharing is not applicable to this article as no datasets were generated or analyzed during the current study.
