# Supplementary material for: The Role of BMAL1 in Regulating Circadian Rhythms During Airway Remodeling in Asthma
Source: J Immunol Res. 2026 Jun 11;2026:6983721. doi: 10.1155/jimr/6983721 (PMC13255153; doi:10.1155/jimr/6983721)
Supplement: Supplementary file 1 — Supporting Information Figure S1: The literature selection flow for this review. A total of 1352 records were retrieved from PubMed, Web of Science, and Scopus. After removing 830 duplicates, 522 records remained. Irrelevant studies were excluded by title screening, leaving 104 records; two additional records were excluded by abstract screening, leaving 102 full‐text articles for eligibility assessment. After further exclusion based on patient, article type, intervention, and full‐text unavailability, 16 reviews and 70 original studies were finally included. [file JIMR-2026-6983721-s001.docx]

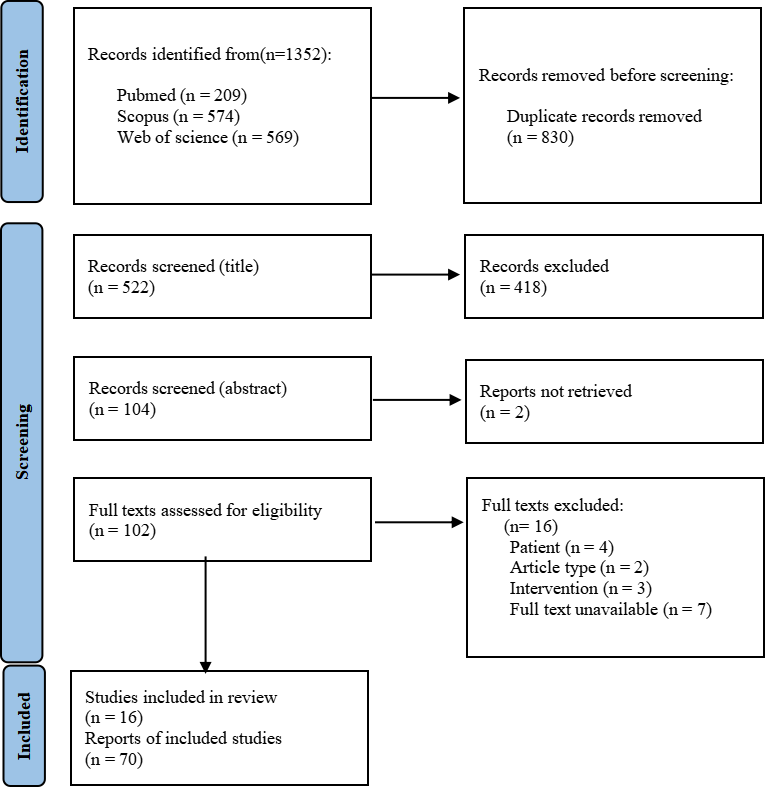


Patient: Non-asthmatic patients.

Article type: Case report.

Intervention: The intervention was irrelevant to circadian clock, BMAL1, or asthma airway remodeling.
